# Role of Daratumumab in Combination With Standard Therapies in Patients With Relapsed and Refractory Multiple Myeloma

**DOI:** 10.7759/cureus.15440

**Published:** 2021-06-04

**Authors:** Anusha Bapatla, Arunima Kaul, Paramvijay Singh Dhalla, Ana S Armenta-Quiroga, Raheela Khalid, Jian Garcia, Safeera Khan

**Affiliations:** 1 Internal Medicine, California Institute of Behavioral Neurosciences & Psychology, Fairfield, USA; 2 Internal Medicine, Detroit Medical Center/Wayne State University/Sinai Grace Hospital, Detroit, USA; 3 Medicine, California Institute of Behavioral Neurosciences & Psychology, Fairfield, USA; 4 Internal medicine, California Institute of Behavioral Neurosciences & Psychology, Fairfield, USA

**Keywords:** daratumumab, relapsed and refractory multiple myeloma, monoclonal antibodies, novel agents, mechanism of action

## Abstract

Multiple myeloma (MM) is a hematological malignancy characterized by renal insufficiency, bone lesions, anemia, and hypercalcemia. In this modern era of medicine, even with the development of drugs like immunomodulatory agents (IMiDs) and proteasome inhibitors (PI), the treatment of MM prevails as a challenge. However, even after the attainment of total remission, relapse of MM and disease progression is frequent. That is why there is an urgent requirement to develop novel monoclonal antibody drugs. The latest drugs for the treatment of relapsed and refractory MM (RRMM) approved by the Food and Drug Administration (FDA) are elotuzumab and daratumumab. In this article, we will discuss daratumumab with different combination therapies. The literature exploration was done using PubMed, Medline, PubMed Central, and Research Gate. Keywords used to search are monoclonal antibodies, daratumumab, RRMM, and novel agents. Our review article, which includes 21 relevant articles, demonstrated that daratumumab in different combinations showed significant progression-free survival (PFS) without severe safety concerns. However, while observing all the studies, neither of them studied the combination therapies of daratumumab in end-stage renal disease (ESRD) patients. Hence, more randomized controlled clinical trials should be done to understand and compare the effect of the combination of daratumumab with the standard of care therapies in ESRD patients.

## Introduction and background

Multiple myeloma (MM) is defined as the abnormal proliferation of plasma cells (PCs) with increased monoclonal antibody production. It is the second common hematological malignancy. MM is characterized by organ dysfunction, including renal insufficiency, anemia, bone lesions, and hypercalcemia. The SEER Data Registry, also known as US surveillance, epidemiology, and end results, estimated that new MM cases in 2021 are 34,920, and estimated deaths in 2021 are 12,410 [1]. Relapsed myeloma is defined as previously treated myeloma that progresses and needs salvage therapy initiation. It does not meet the criteria for either Relapsed and Refractory MM (RRMM) categories or primary refractory myeloma [2]. When the disease is nonresponsive while on salvage therapy or progresses within 60 days of the last cycle in patients who have achieved minimal response or better at some point is defined as RRMM [2]. When the disease is nonresponsive in patients who have never achieved a minimal response and neither got better with any therapy is defined as primary refractory myeloma [2]. Despite enormous advances with the advent of proteasome inhibitors (PI) and immunomodulatory agents (IMiDs), relapse of MM and disease progression is very common even after achieving complete remission. Hence, the treatment of MM remains a challenge [3]. Patients' survival rate with frequent relapses or patients who are refractory to treatment is very low [4]. The median overall survival (OS) in patients with three or more prior therapy lines including an IMiDs or PI or double refractory to a combination of both PI and IMiDs was only about eight months, detailed in a recent retrospective report of real-world survival outcomes [5]. Hence, new treatment combinations and therapies are required urgently in patients with RRMM [4].

In 2015, the Food and Drug Administration (FDA) approved two monoclonal antibodies to treat RRMM, elotuzumab, and daratumumab [6]. Despite the utilization of these novel agents, nearly all patients experience relapsed disease [6], but comparatively prolonged survival with the use of these novel agents. Daratumumab is a human IgGκ monoclonal antibody that targets CD38, expressed in abundance in hematopoietic cell types, especially myeloma cells [7,8]. Daratumumab induces the death of PCs by antibody-dependent phagocytosis, apoptosis, and cell-mediated cytotoxicity. In addition to that, daratumumab works as an immunomodulator by increasing the activity of cytotoxic T‐lymphocytes by inducing and decreasing CD38+ immunosuppressive myeloid and lymphoid cells [9,10].

A pooled observation of two monotherapy studies produced a median OS of 20.1 months and an overall response rate (ORR) of 31%. It showed a strong response and clinical benefit in patients with stable disease responses or better [5]. Based on the above findings, daratumumab was approved in the United States for a monotherapy drug (16 mg/kg) for treatment of MM patients who have received three or more than three prior therapy lines, including a PI and an IMiDs, or who are double‐refractory to a PI and an IMiDs [5]. Daratumumab is extensively studied in various combinations with the standard of care therapies for RRMM. Daratumumab-based combinations showed significant benefit in phase three and phase one/two clinical trials [11-13]. But there is limited data on the complete role of daratumumab in a combination of the standard of care therapies. This article discusses different combination therapies' efficacy and safety, and the use of these combination therapies in different settings.

## Review

We searched the databases PubMed, Medline, PubMed Central, and Research Gate using a combination of keywords. Keywords used to search are daratumumab, RRMM, monoclonal antibodies, and novel agents. The articles included are those from the last five years. Grey literature is not included in this review article. Only articles with free full-text available are included in the study - no location specifications. Only articles in the English language are included.

Mechanism of action of daratumumab 

CD38 is a type II transmembrane glycoprotein involved in numerous intracellular and extracellular functions. CD38 expression is at low levels in the normal lymphoid cell lineage and normal myeloid cell lineage. However, CD38 is expressed at high levels on malignant myeloma cells [8]. Krejick et al., in their flow cytometric analysis on 148 patients, confirmed the increased expression of CD38 on myeloma cells [14]. Daratumumab is an IgG1 human monoclonal antibody against CD38. Daratumumab kills myeloma cells via different pathways include antibody-dependent cellular phagocytosis (ADCP), antibody-dependent cellular cytotoxicity (ADCC), complement-dependent cytotoxicity (CDC), direct apoptosis, and immunomodulatory action. 

Antibody-Dependent Cellular Phagocytosis

ADCP is one of the Fc component-medicated mechanisms that contribute to the anti-tumor activity of daratumumab. Daratumumab (IgG1 monoclonal antibody) binds to CD38; the Fc component of daratumumab binds to FcγRs on the macrophages and then phagocytosis of opsonized tumor cells [15,16]. This mechanism contributing to the anti-tumor activity of daratumumab is well demonstrated by Overdijik et al. in their in vivo study [10]. It is one of the important mechanisms because of macrophages' ability to engulf multiple opsonized tumor cells and the ability to engulf quickly. The efficacy and quick action of macrophage-induced phagocytosis is demonstrated by time-lapse imaging microscopy [10].

Antibody-Dependent Cellular Cytotoxicity

This is one of the Fc component-mediated mechanisms of action. Fc component of daratumumab binds to FcγRs on effector cells. Effector cells mainly release toxic components and thereby results in lysis of the tumor cells [15,16]. Casneuf et al. demonstrated that even though there is a reduction in the number of natural killer (NK) cells as these cells express CD38, there is no demonstrable change in the safety and efficacy of daratumumab. This no change in safety and efficacy is because the remaining NK cells can contribute to ADCC, other granulocytes contribute to ADCC, and other mechanisms of actions also play a role in the efficacy of daratumumab [17].

Complement-Dependent Cytotoxicity

Fc binding to the complement activates complement and results in the production of membrane attack complex, leading to increased permeability of membrane and damage to the cell [16].

Direct Apoptosis

Programed cell death is triggered by cross-linking CD38 by inhibitory FcγRIIb and activating FcγRs [16].

Immunomodulatory Action

**Figure 1:**
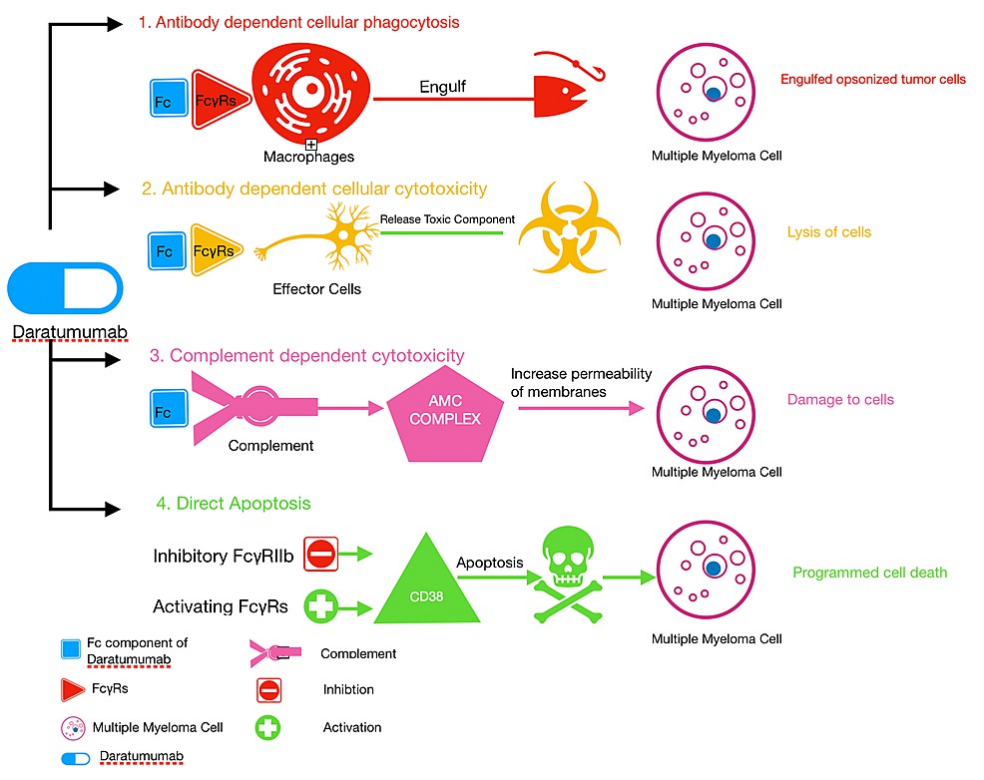
Mechanism of Action of Daratumumab

Increased expression of CD38 is noted on B-regulatory cells and myeloma-derived suppressor cells. As daratumumab is a monoclonal antibody to CD38, these cells are sensitive to daratumumab and hence promote anti-tumor activity. Krejick et al. found that a shift towards positive versus negative immune cells might contribute to anti-tumor activity. But further studies were required to prove whether this T-cell shift results in actual anti-tumor activity or not [14]. The mechanism of action of daratumumab is illustrated in Figure 1.

Modes of resistance 

Resistance pattern to daratumumab may be primary or secondary resistance.

Mechanism of Primary Resistance to Daratumumab

The level of expression of CD38 itself affects the efficacy of daratumumab. MM cells with high levels of CD38 expression are more sensitive to daratumumab than cells with a low level of CD38 expression [15].

Mechanism of Secondary Resistance to Daratumumab

Decreased expression of CD38 following daratumumab infusion causes decreased efficacy. Soluble CD38 and antidrug antibodies might neutralize CD38 theoretically, but no clinical evidence till now [15]. ADCP resistance is mainly caused by Fc receptor polymorphism; low monocyte to myeloma ratio decreases the activity of daratumumab. CD47 expression inhibits phagocytosis of myeloma cells. ADCC resistance is usually caused by increased NK cell destruction, an increased bone marrow stromal cells, and polymorphism of the Fc receptor. CDC resistance is due to CD46, CD55, and CD59 are membrane-associated complement inhibitors that protect normal cells from complement medicated damage. These inhibitors also protect myeloma cells from complement-mediated damage. At the time of progression with daratumumab therapy, increased expression of CD55 and CD59 is shown. Resistance to direct effects is due to a decrease in CD38 expression. Resistance to immunomodulatory action is due to a decrease in activated T cells [16].

Pharmacokinetics of daratumumab

Clemens et al., in their analysis of pharmacokinetics in 223 patients in Gen 501 and SIRUS study, showed that daratumumab exhibits non-linear pharmacokinetic characteristics after intravenous administration. Maximum concentration (C max) increase is in proportion to an increase in dose after initial infusion. After subsequent infusions, the C max increase is greater than the dose-proportional increase. The reason for this increase in C max is postulated either due to target-mediated saturation or due to a decrease in CD38 containing cells by decreasing tumor burden [18]. This study also studied the dose-effect and confirmed that 16 mg/kg is a better target saturation and ORR than 8 mg/kg. Based on the consideration of pharmacokinetic characteristics, the suggested daratumumab dose is 16 mg/kg weekly for eight weeks, every two weeks for 16 weeks, and four weeks after that [18].

Although daratumumab was proven as an effective drug, it is also associated with infusion-related reactions (IRR) in 50% of patients. Most of them are mild and related to early infusions. Usmani et al., in their study, mainly focused on the safety and pharmacokinetics of subcutaneous delivery of daratumumab with hyaluronidase. Two doses were tested in this study, the first dose is 1,200 mg, which is equivalent to the daratumumab IV dose of 16 mg/kg, and the second dose is 1,800 mg, which is equivalent to the daratumumab IV dose of 24 mg/kg. The second dose is tested considering the bioavailability of less than 100% for subcutaneous delivery. Serum daratumumab concentrations in the 1,800 mg subcutaneous group are almost similar to daratumumab 16 mg/kg infusion. Treatment site reactions include pain, erythema, and induration. Most of them were resolved without intervention. IRR is less common than IV infusion. As there are considerably fewer side effects with maintaining efficacy, subcutaneous daratumumab can be considered an alternative [19]. Mechanism of action, modes of resistance and pharmacokinetics are mentioned in Table 1.

**Table 1:** Mechanism of Action, Modes of Resistance, and Pharmacokinetics of Daratumumab MDSC – myeloma derived suppressor cells, B regs – B regulatory cells, T regs – T regulatory cells, NK cells – natural killer cells, ADCP – antibody-dependent cellular phagocytosis, ADCC – antibody-dependent cellular cytotoxicity, CDCC – complement-dependent cellular cytotoxicity, IRR – infusion-related reaction, TEAEs – treatment effective adverse events.

Author & Year of Publication	Number of patients	Type of the study	Purpose of the study	Results	Conclusions
Krejcik et al. 2016 [14]	148	Clinical trial	Role of immunomodulatory effect of daratumumab in RRMM	Immunosuppressive cells, including MDSCs and B regs, showed increased CD38 expression, and daratumumab decreases the number of these cells. T regs, which showed increased CD38 expression, are more immunosuppressive and decreased following daratumumab. Daratumumab effect on T regs also have anti-tumor activity Observed changes were in ratios of CD8:CD4 and CD8: T regs with the treatment of daratumumab. But their exact contribution to the mechanism needs further evaluation and treatment.	Along with the direct effect of daratumumab on myeloma cells, the Immunomodulatory effect of daratumumab on anti-tumor activity is proposed in this study. Further studies were needed to evaluate the change in T cells contributes to the anti-tumor activity of daratumumab or not.
Overdijk et al. 2015 [10]	12	Clinical trial	To study the contribution of macrophage-mediated antibody-dependent phagocytosis to the mechanism of action of daratumumab	To study the macrophage-mediated phagocytosis, double-positive macrophages and percentage reduction in target cells were studied in vivo. Flow cytometry phagocytosis assay showed an increase in double-positive macrophages and reduced the number of target cells. Time-lapse imaging microscopy showed fast and effective phagocytosis by macrophages.	This study demonstrated the contribution of macrophage-mediated ADCP in anti-tumor activity by eliminating ADCC and CDCC. ADCP is one of the fast and effective mechanisms that contribute to daratumumab anti-tumor activity.
Donk et al. 2018 [15]		Review	A review describing different modes of action. It also describes host and tumor-related factors that affect daratumumab efficacy Various mechanisms that cause resistance to CD 38 antibodies	Described mechanism of action as Fc mediated effects includes ADCP, ADCC, and CDCC. Others described are immune effects, direct apoptosis, and Immunomodulatory effects were reviewed. Primary Resistance depends on the level of expression of CD38 on MM cells. Cells with high expression are more sensitive, and cells with low expression are less sensitive. Various factors contribute to secondary resistance. The downregulation of CD38 is another factor that contributes to resistance. CD38 antibodies and soluble CD38 might inactivate but clinically haven't proven that they decrease activity. Divided resistance as ADCP resistance, ADCC resistance, CDC resistance, resistance to direct effects, resistance to immunomodulatory effects	Various host-related and drug-related factors are involved in the therapeutic efficacy of the drug. A better understanding of these factors helps in the individualization of the treatment and thus increases the efficacy
Morandi et al. [16]		Review	Review article on Immunotherapeutic approaches in MM	Along with other immunotherapeutic agents against CD8, this study also reviewed daratumumab. Added an important point of daratumumab in the prevention of bone resorption by killing osteoclasts	This study concludes CD38 as a good target for anti-tumor therapy in MM
Casneuf et al 2017 [17]	148	Clinical trail	Effect of daratumumab monotherapy on NK cells and NK cells dynamics on efficacy and safety of daratumumab	This study demonstrated CD38 mediated reduction in NK cells after daratumumab treatment. In vivo studies showed a decrease in ADCC mediated lysis, and the remaining PBMCs showed some ADCC mediated lysis of cells.	Although there is a reduction in the number of NK cells with daratumumab, no effect on efficacy and safety was observed. There is no effect on efficacy because other effector cells may carry out ADCC, and other mechanisms act.
Clemens et al 2017 [18]	223	Clinical trail	Characterize the pharmacokinetics of daratumumab	This study showed that daratumumab exhibits non-linear pharmacokinetic characteristics after Intravenous administration. C max increase is in proportion to an increase in dose after initial infusion. However, after subsequent infusions, the C max increase is greater than the dose-proportional increase. The reason for this increase in C max is postulated either due to target mediated saturation or due to a decrease in CD38 containing cells by decreasing tumor burden.	The dose regimen suggested from this study is daratumumab 16 mg/kg weekly for eight weeks, every two weeks for 16 weeks, and four weeks after that.
Usmani et al. 2019 [19]	88 patients	Phase one, open-label, multicenter, dose-escalation two-part study	The purpose of the study is to see whether daratumumab can be given SQ without affecting efficacy to minimize complications and discomfort associated with IV daratumumab	Daratumumab concentrations in the 1800 mg Subcutaneous group are almost similar to daratumumab 16mg per kg infusion. One patient was positive for daratumumab antibody, but it seems like it's not affecting pharmacokinetics. Treatment site reactions include pain, erythema, paraesthesia, and induration. Most of them were resolved without intervention. IRR is less common than IV infusion. If present, it easily gets treated with supportive treatment.	Low risk of immunogenicity, low IRR, Low TEAE, and almost equal efficacy are proven with SQ Dara. However, as there are considerably fewer side effects with maintaining efficacy, subcutaneous daratumumab can be considered an alternative.

Combination therapy

Daratumumab monotherapy at a dose of 16mg/kg was studied in Gen 501 and SIRUS study and showed significant ORR in one-third of the population. In addition, treatment efficacy and side effect profile of daratumumab combined with various standards of care regimens in RRMM were studied in different phase clinical trials. Those results from different clinical studies are reviewed here in this article. ​​​​​​

Therapeutic Efficacy of Daratumumab in Different Combinations

Daratumumab with bortezomib and dexamethasone (D-Vd): The CASTOR study is multicenter, randomized controlled trial (RCT). Patients included in the study were the patients with RRMM, those who had at least some positive response to previous therapy, and those who got at least one dose previously. The patients excluded from the CASTOR study had significant side effects to bortezomib or patients who were refractory to bortezomib therapy. They were randomly distributed into two groups. One group got bortezomib and dexamethasone (Vd), and another group got D-Vd. Progression-free survival (PFS) was the primary endpoint that was being studied. After a median follow-up of about eight months, pre-specified interim analysis was done by Palumbo et al. The estimated one-year PFS with the daratumumab group is 60.7% (95% Confidence interval [CI], 51.2 to 69.0) while comparing with the control group, which was 28.8% (95% CI, 17.1 to 37.5). This significant PFS was maintained in all subgroups divided based on age, sex, type of myeloma, number of the previous line of therapy, previous bortezomib therapy, and disease refractory to prior therapy. But the PFS was not studied in subgroups based on cytogenetic risk. The OS benefit was unable to assess as it is a shorter duration of follow-up. From this short-term follow-up published by Palumbo et al., D-Vd is more effective than Vd concerning rates of excellent partial response or better and/or overall complete response or better. This interim analysis showed that D-Vd is superior to Vd in every subgroup of the population was studied [20].

Spencer et al. conducted an updated analysis of the CASTOR study after an extended follow-up of another 12 months (total of 19.4 months). Patients showed significant PFS in the treatment group compared to the control group. An 18-month PFS rate of 48.0% in the D-Vd group compared with the Vd group, which is 7.9%. ORR was significantly improved with D-Vd versus Vd (83.8% versus 63.2%; P<0.0001). This clinical benefit is consistent with all subgroups, including the subgroups based on cytogenetic risk, which was not evaluated in the interim analysis. This extended follow-up showed a deeper response with the D-Vd group with minimum MRD negative rates. OS is still immature to comment on during this study [21]. Mateos et al. did subgroup analysis on efficacy and safety of D-Vd based on age group. Two groups were studied, one is 65 to 74 years, and another is more than or equal to 75. PFS was significantly prolonged in two age groups (age more than 75 and age from 65 to 74). The 18-month PFS in patients with age more than 75 years is 48.0% versus 7.9%, comparable with the 18-month PFS of the updated CASTOR study analysis. Among patients with the age group of 65 to 74, the PFS rate was 45.8% versus 0%, comparable with the 18-month PFS of updated castor study analysis [22].

Daratumumab with lenalidomide and dexamethasone (D-Rd): POLLUX study is a multicenter, RCT, phase three clinical trial. Patients with RRMM, according to the International myeloma workgroup, those who received at least one prior therapy were included in this study. Those who were refractory to lenalidomide or had unacceptable adverse events (AEs) with lenalidomide were excluded from the study. The patients were randomly assigned into two groups. One group received D-Rd, and another group received lenalidomide and dexamethasone (Rd). Pre-specified Interim analysis of this study was done by Dimpolous et al. after a median follow-up of 13.5 months, which showed significant PFS. The time to event analysis of PFS after 12 months was 83.2% in the D-Rd versus Rd is 60.1%. Significant PFS is maintained in all subgroups stratified based on the number of the previous therapy line, whether the patient is exposed to lenalidomide or not, a timeline of previous therapy, and age. ORR in the D-Rd was 92.9% versus 76.4% in the control group. A significantly higher ORR, deeper responses, and longer response duration were shown in this study. The daratumumab group showed a 63% lower risk of death or disease progression than the control group [12].

Dimpolous et al. conducted their updated analysis of the POLLUX study with additional 12 months of follow-up (total of 25.5 months), which showed a significantly reduced risk of progression or death than the control group. A 24-month PFS rate was 68.0% in the treatment group versus 40.9% in the control group. ORR is 92.9% in the D-Rd group versus 76.4% in the Rd group. This study is focused on minimal residual disease and clinically relevant subgroups. Also analyzed parameters in the subgroup analyses were conducted based on the number of prior lines of therapy, prior treatment exposure, and a treatment-free period after the last dose, and cytogenetic risk. The efficacy is maintained in all subgroups, including those with cytogenetically high risk [23]. Bahlis et al. did efficacy and safety analysis after an extended follow-up of 44.3 months. The study showed that the daratumumab combination was shown to cause significant PFS benefit [24]. Mateos et al. did a subgroup analysis of the efficacy and safety of D-Rd based on age group. Two groups are studied 65 to 74 and more than or equal to 75 after an extended follow-up of POLLUX study for 25.4 months. PFS was significantly prolonged in patients aged more than 75 years and patients between 65 and 74 years. In patients aged more than 75 years, the 18-month PFS rate of D-Vd versus Vd was 86.2% versus 36.9%. In patients aged 65 to 74 years, the 18-month PFS rate was 72.0% versus 48.7%. Both the groups showed good ORR and very good partial response (VGPR) or better and CR or better. Mateos et al. demonstrated that efficacy and tolerability are almost the same events in the patents more than 75. So daratumumab can be used even in patients above 75 as the general population [22]. Suzuki et al. performed a subgroup analysis on the East Asian population. This subgroup analysis was performed to check for any change in the D-Rd efficacy and safety in the East Asian population (Japanese, Korean, and Taiwanese). In the East Asian population, 24-month PFS was 65.69% (D-Rd) versus 35.2% (Rd). ORR with the East Asian population was 90.2% versus 72.1 %. ORR with the Japanese population was 90.0% versus 60.0%. This efficacy is maintained in all subgroups, including the cytogenetic group, and consistent with the overall population [25].

Daratumumab with pomalidomide and dexamethasone: Chari et al. discussed the results of the daratumumab plus pomalidomide and dexamethasone (pom-dex) arm from the EQUULEUS study, which includes 103 patients. This study is phase one; an open-label, the non-randomized study focused on dose-limiting toxicities of the daratumumab and pom-dex. This study also focuses on daratumumab and pom-dex safety and tolerability. Those who received at least two lines of therapy were included in this study. The patients were eligible who have progressive disease on lenalidomide or bortezomib or a combination of lenalidomide and bortezomib. The ORR was 60%. ORR was maintained in all subgroups stratified based on various parameters. ORR in subgroups were 64%, 65%, and 55% in patients who received two, three, or more than three lines of prior therapy. Median survival was about nine months. This study showed that pom-dex could be safely combined with daratumumab. This study might complement the POLLUX study. In the POLLUX study, the addition of daratumumab for lenalidomide, which is immunomodulatory, showed significant ORR. The addition of pomalidomide also showed significant ORR, but this is a non-randomized study and needs a very extensive study to prove the combination of daratumumab with pom-dex to be effective. APOLLO study is an on-going study to determine the actual benefit from the RCT. This regimen is approved based on the phase one/two clinical trials without the phase three trial. This regimen is really helpful in people who are refractory to lenalidomide [26].

Hussain et al. did a retrospective cohort study to see the combination of daratumumab and pomalidomide helps overcome the resistance of either of them, considering their mechanism of action. This study showed that a combination of daratumumab and pomalidomide helps overcome resistance in some cases. But this is a retrospective cohort study with a limited population, needs a randomized controlled trial to confirm the association that has been shown in this study [27]. Siegel et al. in their clinical trial mainly focused on patients who are refractory or relapsed with lenalidomide. One-year PFS in those who relapsed after lenalidomide was 83.2%. One-year PFS for those who are refractory to lenalidomide treatment was 72.4%. PFS in patients with one or two prior lines of therapy was 78.8 % versus 69.0%. ORR was achieved irrespective of several prior therapy lines, refractoriness to lenalidomide, or previous bortezomib exposure. These findings demonstrate that pomalidomide, low-dose dexamethasone, and daratumumab combination is a safe and effective treatment for patients with RRMM immediately following disease progression on or after lenalidomide [28].

Daratumumab with carfilzomib and dexamethasone (D-Kd): Safety, pharmacokinetics, and preliminary efficacy from the D-Kd arm of the EQUULEUS were studied by Chari et al. The patients included in this study are RRMM, lenalidomide refractory patients, who had received one to three prior lines of therapy. The 12-month PFS in patients who received D-Kd was 74%. In lenalidomide refractory patients, 12-month PFS was 65%, and bortezomib refractory patients, 12-month PFS was 60%. Median PFS was not reached in all patients. In the treatment group, the 12-month OS rate was 82%. ORR was 84% in lenalidomide refractory patients, and in bortezomib refractory patients ORR was 84%. Median OS was not achieved. The 12-month OS rate for all treated populations was 82%, lenalidomide refractory patients were 75%, and bortezomib refractory patients was 76% as it is a phase one study. Phase three trials are necessary to find out the efficacy of this combination and check the side effect profile. This regimen would be a helpful regimen for those with refractoriness to lenalidomide. There is one study going for D-Kd in lenalidomide-exposed patients [13].

Daratumumab with bortezomib, cyclophosphamide, and dexamethasone (D-VCd): D-VCd was studied in a multicenter, open-label, single-arm study which includes 14 RRMM patients along with 87 patients of NDMM. This study is limited as they involve only 14 patients and the main population in NDMM. The ORR after the completion of four cycles was 71.4 %. Median PFS was 13.3%, and 12-month PFs were 66.2%. 12month OS rate was 54.5%. As this study is a phase two study and no set of controls and a limited number of the population included in this study, it needs an extensive clinical trial to prove the efficacy [29].

Influence of Disease Characteristics, Patient Characteristics, and Other Monoclonal Antibodies in Combination Therapy

Except for the type of myeloma, there is no influence of disease and patient characteristics on the concentration of daratumumab. Daratumumab concentration is less in IgG type compared to non-IgG. Regarding efficacy, there is no change in the efficacy of IgG MM and non-IgG MM. No patient and disease factors analyzed by Yan et al. showed a statistically significant difference [30].

Hoylman et al. did a retrospective cohort study to find out the optimal sequence of daratumumab and elotuzumab. The effect of one monoclonal antibody on another monoclonal antibody was never studied before; ORR to initial monoclonal antibody was unchanged. ORR is higher in the cohort where daratumumab received a second dose compared to elotuzumab as a second dose. PFS for daratumumab when daratumumab first received was about three months. PFS for elotuzumab when elotuzumab first received was about six and half months. Median PFS for daratumumab when received second dose was about ten months. The median PFS for elotuzumab when elotuzumab received the second dose was about two months. Use of daratumumab before elotuzumab was associated with decreased cumulative PFS. Daratumumab retains activity whether it was used as first or second after elotuzumab. But elotuzumab did not retain activity when used after daratumumab [6].

AEs Associated With Daratumumab in Different Combinations

Although AEs associated with a combination of medications are high, most of them are manageable and almost similar to the AEs associated with individual medication involved in combination therapy. In the interim analysis of the CASTOR study, although there were more hematological and non-hematological AEs reported in the D-Vd group, the treatment discontinuation because of side effects is almost similar. No cases of immunogenicity or no cases of hemolysis were reported in the treatment group [20]. Increased incidence of IRR in the daratumumab group and most of them were associated with the first dose of daratumumab infusion. Most of them are grade one or two [20]. Even with an extended follow-up of another 12 months, the safety profile remains unchanged. No new cases were reported [21]. AEs with D-Rd are similar to knowing Rd complications demonstrated in POLLUX study [12]. AEs related to the D-Rd group with extended follow-up are higher than the control group, but the discontinuation rate is low [23]. A higher incidence of infusion reaction is evident in the daratumumab group over 75 years in a study by Mateos et al. [22]. Side effects profiles were similar in both the East Asian and Japanese groups, consistent with the overall population in the study by Suzuki et al. [25]. Though higher neutropenia rates were observed in the daratumumab plus pom-dex group, febrile neutropenia and grade three/four infections are comparable with pom-dex alone [26]. Yimer et al., in their study, show that AEs are more common in RRMM than NDMM in patients receiving D-VCd [30]. Daratumumab therapeutic efficacy and safety as combination therapies are mentioned in Table 2.

**Table 2:** Therapeutic Efficacy and Safety of Daratumumab in Combination Therapies RCT - Randomized controlled trial, D-Vd - daratumumab, bortezomib, and dexamethasone, Vd - bortezomib and dexamethasone  PFS -  progression-free survival, OS - overall survival, ORR - overall response rate, , D-Rd - daratumumab, lenalidomide, and dexamethasone, Rd - lenalidomide and dexamethasone, Pom-dex - pomalidomide and dexamethasone, D-Kd - daratumumab, carfilzomib, and dexamethasone, D-VCd – Daratumumab, bortezomib, cyclophosphamide and dexamethasone, TEAEs - treatment effective adverse events, MRD – minimum residual disease, CR – complete response, VGPR – very good partial response

Author & year of publication	Number of patients	Type of the study	Purpose of the study	Intervention studied	Results/Conclusion	
Palumbo et al. 2016 [20]	498	Phase three, RCT	Effect of D-Vd vs. Vd in patients with RRMM	The primary endpoint is PFS. Secondary efficacy endpoints are ORR, OS, the duration of response, the time of response, time of disease progression, the percentage of people with great partial response	After 12 months in the D-Vd group, the percentage of patients free from disease progression was 65.4%, whereas, in the control group, it was 28.8%. This study showed that the PFS of the D-Vd group was elevated compared with the Vd group, and statistically significant PFS was maintained in all subgroups. Increased complications with D-Vd compared with Vd group.	
Spencer et al. 2018 [21]	498	Phase three, RCT	Efficacy and safety analysis of D-Vd with extended follow up of another 12 months	New PFS with updated analysis in comparison with the primary analysis	The 18-month PFS rate of 48.0% in the study group compared with the control group, which is 7.9%. ORR was significantly prolonged with study group is 83.8 % in comparison with the control group	
Mateos et al. 2020 [22]	539	Phase three, RCT, subgroup analysis of CASTOR and POLLUX based on age group	Subgroup analysis of efficacy and safety of D-Vd and D-Rd based on age group with an extended period of follow-up.	Two age groups studied were one more than 75 years and the second group is the age between 65 to 74 years The Median follow-up for the POLLUX study is 25.4 months, and the CASTOR study is 19.4 months	PFS was significantly prolonged in patients age more than 75 years and patients between 65 and 74 years in POLLUX and CASTOR study In patient’s age, more than 75 years, the 18-month PFS rate of D-Vd versus Vd was 86.2% versus 36.9%. In Patients age 65 to 74 years, the 18-month PFS rate was 72.0% versus 48.7%. In patients age more than 75 years, the 18-month PFS rate of D-Rd versus Rd was 48.0% versus 7.9%. Age more than 75 years, 18-month PFS rate of D-Rd versus Rd were 45.8% versus 0%. In the POLLUX study, both the groups showed good ORR and VGPR or better and CR or better. In CASTOR study showed TEAEs were similar in both groups. Higher Rate of IRR in patients more than 75 years compared to 65 to 74 yrs.	
Dimpolous et al. 2016 [12]	569	RCT	In this study, the effect of combination therapy of D-Rd was being studied in RRMM patients compared to Rd alone	The primary endpoint was PFS The secondary endpoint studied was percentage of patients with the good response or better, ORR, percentage of patients with complete response or better, percentage of patients with results below the threshold for minimal residual disease, studies were time to disease progression, duration of response, time of response, and OS	Treatment group showed a result of 63% reduction outcome in the risk of disease progression or death. It was also seen that there was a significantly higher ORR (92.9 % versus 76.4%) and a significantly higher minimal residual disease.	
Dimpolous et al. 2018 [23]	569	Phase three, RCT	To study efficacy and safety of D-Rd with extended follow-up	The primary efficacy endpoint studied was PFS. Secondary efficacy endpoint studied were MRD, ORR, OS, time of response, Rate of VGPR or better and complete or better	Showed prolonged PFS in agreement with the primary analysis. Significantly improved the ORR (92.9% versus 76.4%) respectively In subgroup analysis based on the number of prior therapies, no difference is found in PFS based on the number of prior therapies one, two, or three. Response to daratumumab group is equal in all independent of cytogenetic status. No change in side effect profile from the primary study reinforce the durable response data.	
Bahlis et al. 2020 [24]	569	Phase three, RCT	Long-term safety and efficacy after a median follow up of 3.5 years	The primary efficacy endpoints were PFS Secondary efficacy endpoints were duration of response, percentage of VGPR or better and CR or better, minimal residual disease, ORR, response time, and OS. Subgroup analysis was done based on the number of lines of therapy, prior treatment with lenalidomide, bortezomib refractoriness, achievement of CR or better MRD has assessed at CR three mths, six months after complete response.	Median PFS with the D-Rd group is 44.5 months versus 17.5 months in the Rd group. D-Rd group showed significant PFS compared to Rd in the subgroup of patients who received at least one prior therapy. Forty-two months PFS rates were 57.3% versus 27.8%. The subgroup of patients who received one to three prior therapies showed significant PFS than the Rd group. For example, 42-month PFS rates were 73.6% versus 59.6%. In the subgroup of prior Lenalidomide therapy and Bortezomib refractory group, significantly prolonged progression-free survival. ORR was significantly higher in the D-Rd group versus the Rd group. ORR was higher among all the subgroups. With extended follow-up, no other safety issues were reported. TEAEs were similar in both treatment and groups. TEAEs leading to treatment discontinuation are pneumonia, pulmonary embolism, and shock.	
Suzuki et al. 2018 [25]	96	Multicenter, open-label, RCT	This study is specially performed to see the efficacy safety of D-Rd in the East Asian population and the specific Japanese population.	The primary endpoint was PFS The secondary endpoint was ORR	Twenty-four months PFS in East Asia population was 65.6 9 in D-Rd group versus 35.2 in Rd group. In Japanese patients was not estimable. ORR in East Asia population, D-Rd versus Rd was 90.2 versus 72.1. In Japanese patients, ORR in D-Rd versus RD was 90.0% versus 60.0%. The response is maintained in all subgroup analyses, including cytogenetic group, prior line of therapy. The side effects profile was similar in both the East Asian and Japanese groups, consistent with the overall population. Safety and efficacy are consistent with the overall population in East Asia and the Japanese population	
Chari et al. 2017 [26]	103	Equlleus - Phase one, open-label, non-randomized study. This study published the arm that received pom- dex with daratumumab.	Equlleus study –on safety and tolerability of various combinations. This arm focuses on adverse effects with patients receiving combination therapy with daratumumab plus Pom-dex	The primary endpoint assessed was the maximum tolerated dose of daratumumab. Secondary endpoints studied were efficacy parameters which include ORR and Rate of complete response.	Though higher neutropenia rates were observed in dartumumab plus pom-dex group, febrile neutropenia and grade three/four infections are comparable with pom-dex alone. Infusion reactions has happened in 50% of patients who were receiving daratumumab. However, most of them were able to manage by slowing the infusion rate or discontinuing the infusion. The ORR was 60%. ORR 64, 65, and 55% in the subgroup of patients received two, three or more than three lines of prior therapy. 12 months PFS rate is 42%. Response with daratumumab with pom-dex is rapid, deep, and durable without any increase in safety issues than pom-dex alone except an increase in the incidence of neutropenia	
Hussain et al. 2018 [27]	19 patients Eight were pomalidomide refractory Three were refractory to daratumumab Eight were refractory to both daratumumab and pomalidomide	Retrospective cohort	The purpose of this study is to see whether the combination of daratumumab and pomalidomide combination helps in overcoming the resistance to either of them or both of them, taking into consideration their mechanism of action.	The endpoints studied are PFS. ORR, CR	ORR was 54.6% in daratumumab or pomalidomide refractory cohort and 12.5% in daratumumab and pomalidomide refractory cohort. CR was 63.6 % in daratumumab or pomalidomide refractory cohort and 50% in daratumumab and pomalidomide refractory cohort. Median PFS was about four months for the daratumumab or pomalidomide refractory group and about four and half months for the daratumumab and Pomalidomide refractory group. This study proposed that daratumumab and pomalidomide's combination helps overcome resistance for either agents or both the agents in some cases. Limitations to his study were high and advising for further studies regarding the use of combination in refractory patients	
Siegel et al. 2020 [28]	112	Phase two, clinical trial	The purpose of this study is to see the effect of daratumumab plus pom-dex in Lenalidomide refractory patients. Three cohorts were studied. Cohort A included patients who were given pomalidomide and low dose dexamethasone In cohort B, pomalidomide, low dose dexamethasone, and daratumumab were given. Cohort C in Japanese people received pomalidomide, low dose dexamethasone, and daratumumab	Major inclusion criteria are those who relapsed or refractory to lenalidomide. Major exclusion criteria are those who received prior pomalidomide or dexamethasone	One-year PFS for those who relapsed after lenalidomide is 83.2%. One-year PFS for those who are refractory to treatment is 72.4%. PFS in patients with one or two prior lines of therapy is 78.8 % versus 69.0%. ORR is achieved irrespective of the refractoriness to lenalidomide, number of prior lines of therapy, or previous bortezomib exposure This combination is approved based on these results exhibit that pomalidomide, low-dose dexamethasone, and daratumumab are effective and a safe treatment modality for RRMM patients just after the disease progression on/after lenalidomide.	
Chari et al. 2019 [13]	85	Phase one, open-label, non-randomized study	Safety, pharmacokinetics, and preliminary efficacy of D-Kd	PFS and ORR in lenalidomide refractory and bortezomib refractory patients	In lenalidomide refractory patients, 12 months PFS was 65%, and bortezomib refractory patients, 12-month PFS was 60%. Mainly in lenalidomide refractory patient efficacy is maintained, and this regimen would be a helpful regimen for those with refractoriness to lenalidomide.	
Yimer et al. 2019 [29]	14	Multicenter, Open-label Phase two study	The purpose of this study is to study about efficacy and safety of the daratumumab in combination with bortezomib, cyclophosphamide, and Dexamethasone (D-VCd)	The primary endpoint studied is the rate of complete response plus VGPR in the RRMM	The study shows that TEAEs are more common in RRMM than NDMM.	
Yan et al. 2017 [30]	223	Exploratory analysis	To study the effects of disease characteristics and patient characteristics on daratumumab pharmacokinetics, efficacy, and safety.	Combined data from SIRUS and GEN501 were collected. Disease factors are type of myeloma, prior lines of therapy, refractory status, ECOG performance status at baseline. Patient factors are age, race, sex, body weight, renal and hepatic function.	ORR was similar to both IgG and non-IgG myelomas. This study concluded that none of the factors studied showed statistically significant differences.	
Hoylman et al. 2019 [6]	19 patients. Eight were pomalidomide refractory. Three were refractory to daratumumab Eight patients were refractory to both daratumumab and pomalidomide	Retrospective cohort. Two cohorts were included in this study. Cohort one received daratumumab before elotuzumab Cohort two received elotuzumab before daratumumab	To find out the optimal sequence of daratumumab and elotuzumab. The effect of one monoclonal antibody on another monoclonal antibody was never studied. This study to see any effect of daratumumab and elotuzumab on each other.	The primary outcome looked at was cumulative PFS. Secondary outcomes were PFS for the first monoclonal antibody, PFS for the second monoclonal antibody.	Efficacy outcomes – In both the cohort, ORR to the initial monoclonal antibody was unchanged. The ORR in the cohort receiving the second dose of daratumumab was higher than elotuzumab as the second dose. PFS for daratumumab when daratumumab first received was about three months. PFs for elotuzumab when elotuzumab first received was about six and half months. Median PFS for daratumumab when received second dose was about 10 months. The median PFS for elotuzumab when elotuzumab received a second dose was about two months. Use of daratumumab before elotuzumab was associated with decreased cumulative PFS. Daratumumab retain activity irrespective of whether it was used as first or second after elotuzumumab but elotuzumab did not retain activity when used after daratumumab	
Cejalvo et al. 2019 [31]	15	Retrospective study	Retrospective study of safety and efficacy of daratumumab among patients with RRM and end-stage renal disease requiring dialysis	The primary endpoints studied were ORR, PFS, and OS		ORR was 40% that included one CR plus four VGPR. Median PFS was about nine months. OS was 12.19. Hematological TEAEs observed were anemia, thrombocytopenia, neutropenia and non-hematological TEAEs were asthesia and hypotension. TEAEs causing death in this study were pulmonary infection, gastrointestinal hemorrhage, and ventricular fibrillation. The best treatment regimen for RRMM for patients with end-stage renal disease requiring hemodialysis was single-agent daratumumab.

Our study has some limitations; only articles in the English language and the free text available articles are included. Most of the studies are performed on Caucasians and African Americans but not on Asians except the study by Suzuki et al. on the East Asian population. Patients in these studies are followed for a limited time and need more follow-up to assess OS rate. There are no RCTs found on daratumumab use as combination therapies in end-stage renal disease (ESRD) patients, which is an important association with MM. Only one study by Cejalvo et al. did a retrospective analysis that showed a single agent is safe in ESRD patients. However, this study has limitations; this is a small observational retrospective study [31]. RCTs are necessary to assess the safety and efficacy of single-agent and combination therapies.

## Conclusions

The main objective of this research article is to study the safety and efficacy of daratumumab with the current standard regimes for MM. D-Vd compared to Vd exhibited prolonged PFS in interim and long-term follow-up data. While observing the extended follow-up, all subgroups maintained the prolonged PFS including a subgroup based on cytogenetic risk. Prolonged PFS was observed in the interim and extended follow-up in the D-Rd group. While considering all subgroups (including high cytogenetic risk), notable prolonged PFS was maintained on the D-Rd group compared to the Rd group. Therefore, D-Vd and D-Rd were approved for the treatment of RRMM. During phase ½, clinical trial efficacy and safety were observed with daratumumab plus pom-dex, especially useful in patient's refractory to lenalidomide. During phase one clinical trials, D-Kd and D-VCd also showed promising efficacy. More RCTs are necessary to prove the efficacy and safety of D-Kd and D-VCd. While observing all the studies, none of them mentioned the combination therapies of daratumumab for ESRD patients. Hence, more RCTs are necessary to understand and compare the effect of combinations of daratumumab with the standard of care therapies in ESRD patients.
